# Modulation of Hepatocarcinoma Cell Morphology and Activity by Parylene-C Coating on PDMS

**DOI:** 10.1371/journal.pone.0009667

**Published:** 2010-03-16

**Authors:** Nazaré Pereira-Rodrigues, Paul-Emile Poleni, Denis Guimard, Yasuhiko Arakawa, Yasuyuki Sakai, Teruo Fujii

**Affiliations:** 1 CIRMM, Institute of Industrial Science, University of Tokyo, Komaba, Meguro-ku, Tokyo, Japan; 2 LIMMS/CNRS-IIS, Institute of Industrial Science, University of Tokyo, Komaba, Meguro-ku, Tokyo, Japan; 3 Institute of Industrial Science, University of Tokyo, Komaba, Meguro-ku, Tokyo, Japan; Virginia Tech, United States of America

## Abstract

**Background:**

The ability to understand and locally control the morphogenesis of mammalian cells is a fundamental objective of cell and developmental biology as well as tissue engineering research. We present parylene-C (ParC) deposited on polydimethylsiloxane (PDMS) as a new substratum for *in vitro* advanced cell culture in the case of Human Hepatocarcinoma (HepG2) cells.

**Principal Findings:**

Our findings establish that the intrinsic properties of ParC-coated PDMS (ParC/PDMS) influence and modulate initial extracellular matrix (ECM; here, type-I collagen) surface architecture, as compared to non-coated PDMS substratum. Morphological changes induced by the presence of ParC on PDMS were shown to directly affect liver cell metabolic activity and the expression of transmembrane receptors implicated in cell adhesion and cell-cell interaction. These changes were characterized by atomic force microscopy (AFM), which elucidated differences in HepG2 cell adhesion, spreading, and reorganization into two- or three-dimensional structures by neosynthesis of ECM components. Local modulation of cell aggregation was successfully performed using ParC/PDMS micropatterns constructed by simple microfabrication.

**Conclusion/Significance:**

We demonstrated for the first time the modulation of HepG2 cells' behavior in relation to the intrinsic physical properties of PDMS and ParC, enabling the local modulation of cell spreading in a 2D or 3D manner by simple microfabrication techniques. This work will provide promising insights into the development of cell-based platforms that have many applications in the field of *in vitro* liver tissue engineering, pharmacology and therapeutics.

## Introduction

In tissue engineering, it is essential to know how cells interact with the extracellular matrix (ECM) *in vitro* and translate information received from these extracellular components into an intracellular event [1]–[6]. Mechanisms by which the ECM can affect cell behavior have been intensively described. As the ECM components interact physically with the cell to regulate directly cell functioning *through* receptor-mediated intracellular signaling pathways. Besides, the mechanotransduction of environmental stimuli can be indirectly modulated by the ECM by controlling the mobilization or release of trophic factors (cytokines, growth factors) involved in the switch between cell proliferation and differentiation [7], [8]. Due to the complex role of these interactions in regulating many physiological processes, novel approaches dedicated to controlling ECM structure on culture substratum are required to better understand how the microenvironment influences cell morphology and activity. Therefore, it is of first importance to develop future standardized tissue-engineered platforms that take into account the sourcing of cells and tissues [9].

Different strategies dealing with the modulation of the surface properties of an underlying substratum (hydrophobicity, stiffness, charge, roughness, and so on) were previously reported [10]–[16]. Such surface modulations were shown to induce changes in the supramolecular structure of the adsorbed ECM, leading to differences in cell adhesion and morphogenesis by altering the balance between cell-cell and cell-ECM interactions. These studies demonstrated the importance of materials science in modern tissue engineering, but were limited to the influence of cell substratum surface properties on the initial cell spreading. Moreover, such studies did not focus on the possibility that cells might respond to mechanical environments by remodeling their matrix (coated and/or new synthesized) or by regulating the balance between cell-cell as well as cell-substratum interactions in a different way. Consequently, these surface modifications could not sufficiently mimic the *in vivo* microenvironment due to the fact that an individual cell's response to *in vivo* conditions is regulated by local spatio-temporal cues, such as those provided by the ECM, neighboring cells, soluble factors, and physical forces [1], [3], [16].

New tools based on microfabrication technology and materials science, including surface micropatterning are now used to fabricate advanced engineered products to control substratum surface chemistry and topology, including heterogeneous surfaces that offer precise control of cellular organization and microenvironment [2], [4]–[6], [17]–[20]. To examine how cells react to their environment, the use of new biomaterials and microfabrication approaches presents new challenges. These novel techniques will ultimately facilitate studies of how environmental cues propagate through cell populations, providing insight into development and other morphogenetic processes.

In this study, we focused on modulating cell activity and morphology using two relevant materials widely used in BioMEMS fabrication: polydimethylsiloxane (PDMS) and poly-chloro-p-xylene or parylene-C (ParC). PDMS is a flexible, thermoplastic and transparent polymer that can be easily molded and that has been widely used in the fabrication of biomedical microdevices. PDMS biocompatibility and high reliability provide the great advantage to work safety in the design realization of precise 2D or 3D devices [21]. Parylene is the tradename for a variety of chemical deposited poly(p-xylylene) polymers used as moisture barrier and electrical insulator. Consequently, ParC has been used intensively as a coating material for insulating implantable biomedical devices (stents, defibrillators, pace makers and other devices permanently implanted in the body) [22], [23]. It can be easily vapor-deposited onto substrates to generate highly conformal transparent and homogenous coatings, and can be subsequently micro-patterned without temperature load of the substrate as coating takes place at ambient temperature [24]. Recently, the compatibility of ParC membranes with in vitro cell culture was studied by analyzing protein adsorption and initial cell adhesion to the polymer [24]. However, these results were preliminary and did not address the issue of *in vitro* cell morphogenesis.

In the present work, we demonstrate the suitability of ParC for long-term cell culture and its potential to modulate cell activity and morphology when evaporated or micropatterned onto PDMS. ECM architecture was characterized by atomic force microscopy (AFM), which revealed that differences in ECM structure between PDMS and ParC/PDMS led to disparate hepatocarcinoma cell adhesion, spreading, and reorganization in two- or three-dimensional structures by ECM neo-synthesis. Cell morphological changes were shown to directly influence metabolic activity as well as the expression of liver transmembrane receptors involved in cell adhesion and cell-cell contact. Taken together, the data confirm the potential of using microsized ParC patterns onto PDMS substratum to precisely and locally modulate cell aggregate morphology.

## Results and Discussion

### Substratum Intrinsic Physical Features

Is is well recognized that changing the intrinsic properties (chemical and physical) of an underlying substratum can have an influence on its interactions with proteins, and as a result on its interactions with cells [12], [14]–[16], [25], [26]. To the best of our knowledge, no investigation has been reported on the physical features ParC-coated PDMS (ParC/PDMS) substratum as well as on the impact of this biomaterial on adsorbed type-I collagen topography. In the present paper, we investigated the impact of ParC/PDMS and PDMS on the structure of type-1 collagen structure, when used as cell culture substrata.

ParC/PDMS, PDMS and conventional polystyrene (PS) dish substrata were first exposed to O_2_ plasma and then coated with type-1 collagen overnight before their subsequent analysis. Same surface treatments were used for all substrata before cell culture.

We first examined the wettability of “collagen-untreated O_2_-plasma and/or collagen-treated O_2_-plasma” PDMS and ParC/PDMS (Table 1). PDMS and ParC/PDMS were initially hydrophobic with contact angles of ∼105° and ∼90°, respectively. As previously shown [24], the two substrata also exhibited similar levels of hydrophilicity after type-I collagen treatments. Additionally, the hydrophobicities of PDMS and ParC/PDMS were comparable before (contact angles of 27° and 37° for PDMS and ParC/PDMS, respectively) and were identical after (contact angle of ∼20°) collagen coating.

**Table 1:** Contact angle measurements (θ) of PS, PDMS, and ParC/PDMS substrata before and after surface treatment.

Contact Angle (θ,°)			
*Substratum*	*Untreated*	*O_2_- plasma treated*	*O_2_ plasma + type-I collagen treated*
PDMS	104.2±2.5	26.8±1.4	20.8±3.0
ParC/PDMS	88.4±1.0	36.6±4.4	21.4±3.7

Data are represented as mean of thirty values obtained from three separate experiments.

**Figure 1:**
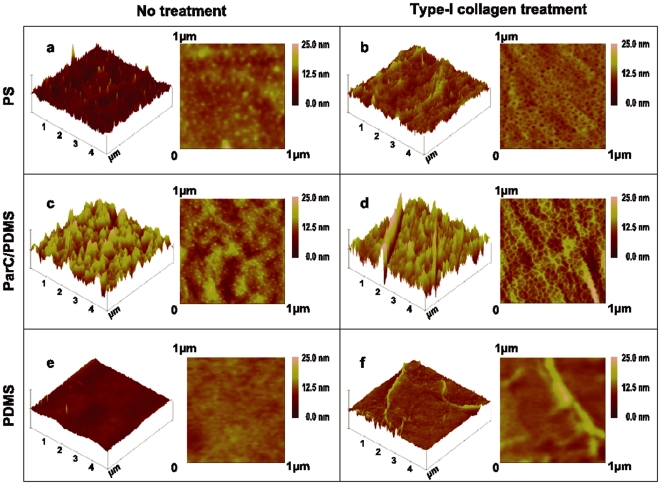
Substratum topography before and after collagen treatment. 5 µm×5 µm and 1 µm×1 µm representative surface topographies (n = 3 per condition) of PS, Par-C/PDMS, and PDMS substrata coated with type-I collagen (b, d & f). Untreated substrata were analyzed as controls (a, c & e).

We next analyzed the substrata roughness by acquiring atomic force microscopy (AFM) topographic images of PDMS, ParC/PDMS, and PS substrata before and after type-I collagen adsorption (Fig. 1). Our results show that collagen adsorbed on ParC/PDMS and PS exhibited a dense network of microstructures around ∼1 nm high and ∼30–50 nm wide, whereas collagen adsorbed on PDMS had a low surface density of elongated fibrils that were ∼2 nm thick and ∼200 nm wide. These microstructures were completely absent from bare substrata. The values of the surface root-mean-square roughness (R_rms_) for all substrata, with or without collagen coating, are listed in Table 2. These data demonstrate that untreated ParC/PDMS and PS substrata are significantly rougher (R_rms_  = 4.7 nm and 1.5 nm, respectively) compared to PDMS before collagen coating (R_rms_  = 0.4 nm). Therefore, ParC deposition onto PDMS induces higher surface roughness compared to PDMS alone, without changing the hydrophobicity of the substratum after type-I collagen coating.

**Table 2:** Surface roughness measurements (R_rms_) of PS, PDMS, and ParC/PDMS substrata before and after surface treatment.

Root-Mean-Square Roughness (R_rms_, nm)		
*Substratum*	*O_2_- plasma treated*	*O_2_ plasma + type-I collagen treated*
PS	1.5±0.1	2.2±0.4
PDMS	0.4±0.1	0.9±0.2
ParC/PDMS	4.6±0.3	7.1±0.9

Data are represented as mean of nine values obtained from three separate experiments.

Recent studies determined that a change in the hydrophobicity of an underlying substratum affected type-I collagen organization [12], [25] and that the substratum roughness was an important parameter that affected the mobility of adsorbed ECM polymers and their tendency to aggregate [13]. In our study, decoupling the impact of wettability and roughness was difficult, as both of these properties changed before and after type I-collagen coating. However, we could observe that roughness changes before and after type-I collagen coating were more significant than wettability changes, suggesting a negligible impact of the wettability on type I collagen microstructuration.

It was previously shown that a substratum with larger surface roughness provides a greater surface area for protein adsorption [25]. Moreover, a transition from a collagen fibril-like structure to a smooth collagen layer was shown to occur when substrata exhibited vertical topographic variations smaller than the collagen molecular thickness (around 1 to 1.5 nm) [27]. Consequently, the present study might suggest that collagen molecules may be relatively free to move on smooth surfaces like PDMS (R_rms_  = 0.4 nm) to form supramolecular microfibril aggregates whereas rougher substrata like ParC/PDMS and PS (R_rms_  = 4.7 nm and 1.5 nm, respectively) inhibit collagen mobility.

### Effects of the intrinsic physical features on HepG2 cells behavior

Considerable efforts have been done in the field of hepatic tissue engineering to obtain a near-term solution to the hepatocyte shortage [28]. To achieve this goal, evaluating the potency of several cell types from liver tissue (primary hepatocytes and hepatocarcinoma) would be a relevant challenge in the field of liver engineering. However, subtle differences in the metabolic response between animal and human hepatocytes are known to limit the performance of a tissue-engineered construct based on animal cells [29], suggesting that the most suitable cell source for hepatic engineering would clearly come from a human-derived source of hepatocytes. HepG2 cells derived from metastatic tumors present an unlimited capacity for growth as well as some normal hepatic functions *in vitro* [30]. For these reasons, this cell line was chosen here in order to evaluate the influence of the intrinsic physical features of ParC/PDMS and PDMS on their morphology, viability and activities.

In order to mimic the *in vivo* liver ECM microenvironment, cells can be cultured on surfaces coated with several types of matrix (Matrigel Matrix, Puramatrix, fibronectin or type I collagen). In the present work, we utilized type-I collagen because it is the most used for liver tissue engineering applications since this matrix enables the culture of hepatic cells (hepatocarcinoma cells as well as fresh or cryopreserved primary hepatocytes) that are capable to maintain their biological activities [31], [32].

### Comparison of HepG2 Cell Viability and Morphology

We first investigated the impact of the observed differences in type-I collagen architecture on HepG2 cells morphology and viability.

**Figure 2:**
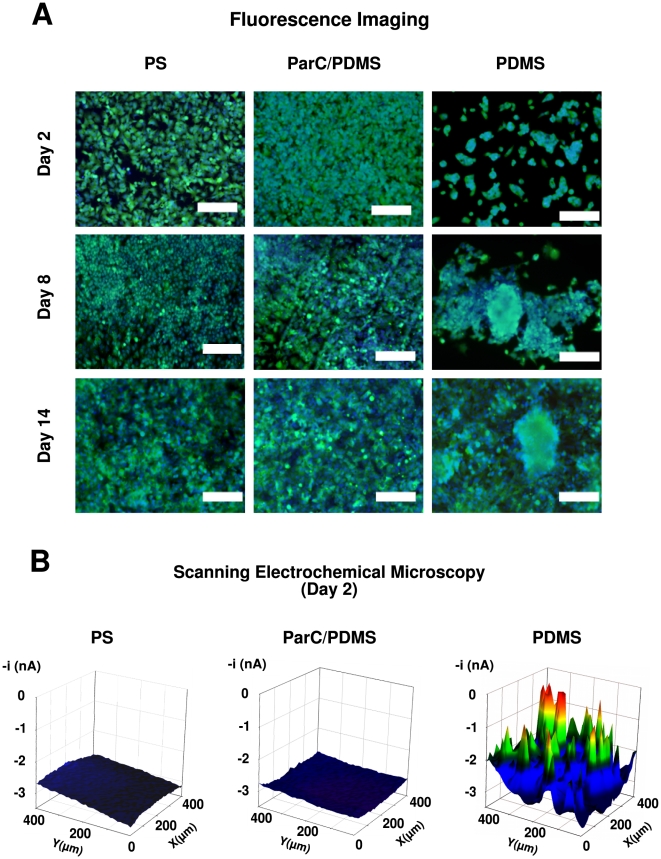
HepG2 cell viability and morphology. **A**) Fluorescent micrographs of HepG2 cells cultured on PS, ParC/PDMS, or PDMS substrata after staining with Calcein-AM (green) and Hoechst (blue) fluorescent dyes on Day 2, 8, or 14. Scale bar  = 200 µm. **B**) Cell topography of HepG2 at Day 2 cultured on PS, ParC/PDMS, or PDMS substrata recorded using FeCN_6_^4-^ oxidation potential for SECM (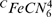 = 5 mmol/L, E_applied_ = 0.5 V vs. Ag/AgCl). Scan area  = 400×400 µm.

Cell viability assays using the fluorescent dyes Calcein-AM (green) and Hoechst 33342 (blue) show that HepG2 cells cultures on PDMS, ParC/PDMS, and PS were viable for up to 14 days and could properly attach to all substrata without any sign of cell death (Fig. 2A). Morphological differences in cell aggregation on PDMS and ParC/PDMS were observed beginning 48 h after seeding. PDMS substratum induced three-dimensional cell aggregation, whereas two-dimensional cell structures were obtained on both ParC/PDMS and PS substrata.

HepG2 cell culture topographies were measured by scanning electrochemical microscopy (SECM) on all substrata in order to quantitatively evaluate the height of early-stage cell aggregates and confirm the observed cell morphological differences between PDMS and ParC/PDMS substrata at day 2. SECM is a scanning probe technique that has the advantage of being non-invasive and is adapted to the topographic analysis of “soft” objects, such as cells by using an hydrophilic redox mediator that does not penetrate the cell membrane [33]–[35]. SECM analysis was chosen instead of conventional histological cross-sections of cells, which may be time-consuming to prepare and difficult to analyze, especially in the case of cell aggregates that can detach during sample treatment. All details concerning the experimental protocol and the principles of SECM measurement are described in Figure S1.

Cell culture topography observed by SECM at day 2 (Fig. 2B) showed that no significant change in tip response was recorded while scanning cells cultured on ParC/PDMS or PS substrata. On the contrary, a significant change in the scanning tip signal was noted for cells cultured on PDMS substratum. This was due to the presence of three-dimensional cell aggregates that partially blocked the diffusion of the redox mediator toward the electrode [34]. By correlating the measured current at the tip to the distance between the scanning tip and the cells (see approach curve in Fig. 1 of SI), we determined that cells grown on PDMS exhibited 13 to 15 µm-high cell aggregates after two days of culture, whereas homogeneous 2 to 3 µm-high cell layers were detected in the case of cells seeded on ParC/PDMS or PS. Therefore, ParC coating onto PDMS was shown to inhibit the formation of the three-dimensional cell aggregates (*i.e.* pre-spheroids) observed in the case of PDMS substratum and to promote a two-dimensional cell culture organization (*i.e.* monolayer).

The mechanisms that lead to such a change in aggregate structure were previously reported for hepatocarcinoma cells and hepatocytes [36]–[38]. It was shown that hepatocyte aggregate morphology was mainly governed by a balance between cell contraction forces and cell-surface adhesion forces. When cell-surface adhesion forces were relatively weak in comparison with cell contractile forces, the cells reorganized into spheroids, while only monolayers were reported when cell-surface forces were not overcome by cell contractile forces. Based on this model of cell aggregation, our results indicate that ParC increases interactions between cells and the substratum as compared to PDMS alone, leading to stronger substratum-cell adhesive forces than cell contractile forces. Moreover, our observations suggest that the balance of these two forces was comparable for ParC/PDMS and PS under our experimental conditions.

We point out that we varied the collagen working solution concentration between 0.06 mg/ml and 0.6 mg/ml (data not shown), while the other experimental parameters for collagen deposition were kept identical. However, the AFM measurements showed no significant difference in either surface roughness or collagen topography (dense network of microstructures for ParC/PDMS and supramolecular and microfibril aggregates for PDMS) with varying solution concentration (data not shown). Accordingly, we did not observe any change in cellular morphology (2D for ParC/PDMS and 3D aggregates for PDMS) (data not shown), suggesting that the obtained data are reliable and poorly sensitive to the conditions of preparation for each substratum, which is advantageous compared to other chemical surface modifications.

Finally, we point out that that parameters other than roughness, such as for instance the chemical impact of ParC or the wettability, might influence the observed collagen microstructuration. However, difference in wettability between substrates is not as significant as that found in roughness. Besides, the collagen deposition process is based on passive adsorption, and thus likely to be more dependent on preparation conditions of the collagen solution (concentration, temperature), rather than surface chemistry.

### Comparison of Cell Activity and Microenvironment

We next analyzed the influence of PDMS, ParC/PDMS, and PS substrata on cell activities and interaction with the surrounding microenvironment.

**Figure 3:**
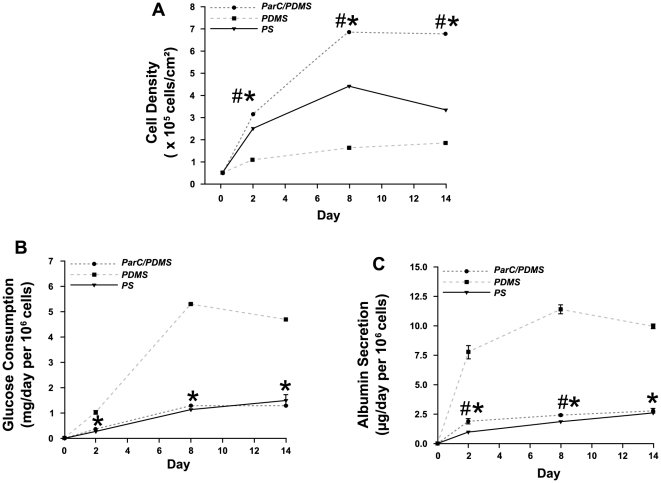
HepG2 cell proliferation and metabolic activity. **A**) HepG2 cell surface density, (**B**) normalized glucose consumption, and (**C**) normalized albumin secretion for up to 14 days of culture on PS (--▴--), ParC/PDMS (--•--), or PDMS (--▪--) substrata. Data are expressed as mean of three to six normalized values obtained from three separate experiments.

On the one hand, cell proliferation (Fig. 3A), glucose consumption (Fig. 3B), and albumin secretion (Fig. 3C) were evaluated for up to 14 days of culture on all substrata. Albumin secretion and glucose consumption were chosen as indicators of hepatic cellular phenotype, as previously reported [38]–[40].

Overall, our data suggest that drastic differences on cell proliferation and albumin secretion occurred between PDMS and ParC/PDMS by 48 h of cell culture whereas no significant differences on cell adhesion was noted after cell seeding. Cell density on ParC/PDMS was 3-fold higher than on PDMS after two days of culture (Fig. 3A), whereas glucose consumption and albumin secretion, which were normalized to 10^6^ cells, were found to be 3-fold to 4-fold lower (Fig. 3B and 3C). These trends in proliferation and metabolic activity were maintained for up to 14 days of culture. Interestingly, cells grown on ParC/PDMS exhibited a higher density compared to those cultured on PS (+25% at day 2 and +100% at day 8 and day 14) and reached a stable level by day 8, whereas cells started to detach on PS substratum from day 8 (Fig. 3A). Additionally, the metabolic activity of cells grown on ParC/PDMS was significantly higher than that of cells grown on PS between day 2 and day 8 (+31% at day 2 and +14% at day 8 for glucose consumption; +100% at day 2 and +30% at day 8 for albumin secretion) until becoming comparable on day 14.

Previous studies demonstrated the correlation between cell morphology and metabolic activity *in vitro* [38], [41]–[43]. It was shown that hepatocytes in spheroid-like aggregates exhibited a differentiated, non-proliferative phenotype while cells in monolayers were more likely to proliferate and less likely to differentiate. These findings supported the hypothesis that three-dimensional aggregates of hepatocytes may serve liver-specific functions.

Our results show that the presence of ParC coated onto PDMS induces a transition from a differentiated to a more proliferative state in HepG2 at early stages of cell culture. Moreover, we show that our chosen experimental conditions (O_2_-plasma treatment followed by overnight collagen coating) allow long-term culture of HepG2 on PDMS and ParC/PDMS, whereas cells' attachment on PS substratum decreased after 1 week of culture.

**Figure 4:**
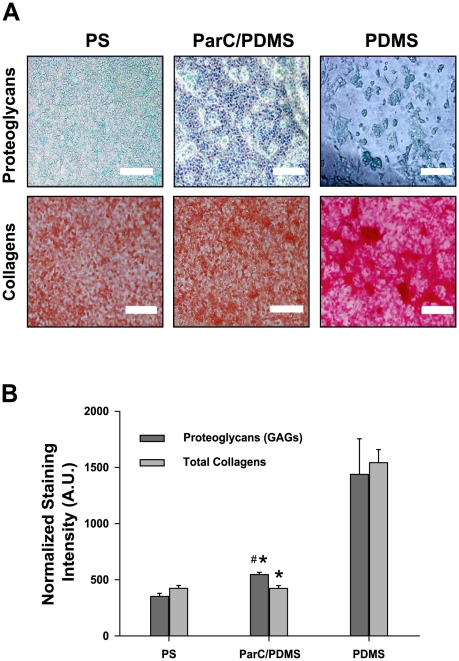
ECM component analysis. **A**) Phase contrast light micrographs of HepG2 cells exposed to Alcian blue and Sirius red for total proteoglycan and collagen synthesis analysis, respectively, after two days of culture on PS, ParC/PDMS, or PDMS substrata (n = 5 per condition). Scale bar  = 50.0 µm. **B**) Staining intensities normalized to 10^6^ cells for each substratum. Data are expressed as mean of five normalized values obtained from three separate experiments.

HepG2 cell-synthesized new proteoglycans and collagens in the ECM were investigated with Sirius red and Alcian blue staining assays after 2 days of culture to put in evidence that the ability of HepG2 cells to maintain their 3D aggregating structure might be due to neosynthesis of new ECM components (Fig. 4A). All staining intensity values were normalized per 10^6^ cells (Fig. 4B). Our results suggest that, after two days in culture, the higher amount of ECM newly synthesized by cells cultured on PDMS compared to on ParC/PDMS (3.5-fold higher proteoglycans synthesis and 5-fold higher collagens) was related to the early three-dimensional rearrangement of the cells. Thus, HepG2 grown on PDMS synthesized higher quantities of ECM at an early stage of culture to avoid cell detachment or death, contrary to cells grown on ParC/PDMS or PS. Due to their more stable organization into a monolayer, the latter cells may not have needed to synthesize additional ECM to the same extent [44]–[47]. This finding is in good agreement with our data obtained on collagen synthesis. Interestingly, we found that HepG2 cells grown on ParC/PDMS synthesized 1.56-fold higher proteoglycans, compared to PS. Since ECM is critically important to such biomechanical properties as cell adhesion, proliferation, morphogenesis, and differentiation [8], [48], [49], HepG2 cells cultured on PDMS may be more metabolically active and serve liver-specific functions. It has also been shown that proteoglycans induce gap junctions in hepatocytes [12], [50], [51], indicating a further role of the ECM in enhancing spheroid cell aggregates' activity. Therefore, our results show that cells grown on PDMS present enhanced hepatic function by providing a more three-dimensional microenvironment. Meanwhile, the presence of ParC may induce a different microenvironment that leads to divergent cell metabolism and cell-cell interactions.

On the other hand, potential differences in the microenvironment established by PDMS and ParC/PDMS substrata were underlined. As cell culture and more particularly albumin secretion were rapidly stabilized, we focused our investigation on the impact of ParC on the expression of representative liver transmembrane receptors in early stage of cell culture, in that case after two days of culture.

Cell transmembrane receptors, such as integrins and cadherins, are known to mediate adhesive cell-ECM interactions and cell-cell interconnection. Integrins physically connect the Arg-Gly-Asp (RGD) sequence of ECM components to the cell cytoskeleton and are involved in the transmission of mechanical signals through this molecular bridge [52]–[55]. β1-integrin and α6-integrin were specifically chosen in this study for their implication in hepatocyte-ECM interactions [56]. Both E-cadherin and Junctional Adhesion Molecule (JAM)-A are acknowledged to influence cell morphology and cell-cell interactions and are closely correlated with the synthesis of biliary pseudo-canaliculi in the case of hepatic cells [57]–[61]. Modulation of the expression of these membrane receptors through cell-ECM interactions thus affects mechanical forces within the cell and results in changes in cell aggregate morphology. Such morphological change would in turn influence the activation of specific intracellular signaling pathways vital to cell survival, migration, and spreading, which in turn influence cell proliferation and differentiation [55], [62], [63].

**Figure 5:**
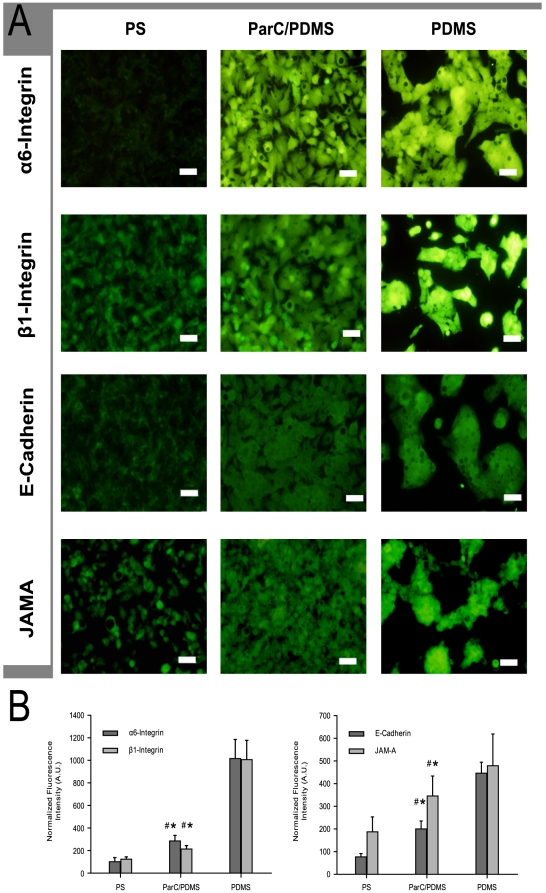
Cell transmembrane receptor expression. **A**) Fluorescence micrographs of β1- and α6-integrin, and E-cadherin and Junctional Adhesion Molecule (JAM)-A expression after two days of culture of HepG2 on PS, ParC/PDMS, or PDMS substrata. Bare scale  = 50 µm. **B:** Fluorescence intensities normalized to 10^6^ cells, of β1- and α6-integrin and E-cadherin and Junctional Adhesion Molecule (JAM)-A expression after two days of culture on PS, ParC/PDMS, or PDMS substrata. Data are expressed as mean of nine normalized values obtained from three separate experiments.

In the present study, we arbitrarily assigned β1-integrin, α6-integrin, E-cadherin and JAM-A as the representative transmembrane receptors that have an important role in either ECM-cell and cell-cell interaction or hepatocytes differentiation. The expression of those proteins was analyzed by immunofluorescence with fluorescence intensities normalized to 10^6^ cells (Fig. 5A and 5B). By 48 h, β1- and α6-integrin levels were found to be markedly enhanced in the PDMS condition as compared to ParC/PDMS (5-fold higher for β1-integrin and 3.5-fold higher for α6-integrin) (Fig. 5B). Similarly, E-cadherin and JAM-A levels were 2-fold and 1.3-fold higher on PDMS than on ParC/PDMS, respectively (Fig. 5B). Higher expression of integrins, E-cadherin and JAM-A on PDMS might be correlated with its higher levels of ECM synthesis, discussed earlier, suggesting that the substratum enhances cell-ECM adhesion and cell-cell interconnections (GAP junctions for E-cadherin and tight junctions for JAM-A). Additionally, the enhanced membrane receptor expression observed in the case of cells cultured on PDMS may be explained by an enhanced ECM turnover (*i.e.*, faster ECM anabolism and catabolism), as previously demonstrated [27], [64].

Interestingly, the expression of representative liver plasma transmembrane proteins was higher in the case of ParC/PDMS, as compared to PS (3-fold and 1.6-fold higher for α6- and β1-integrins, respectively; 2.5-fold higher and 2-fold higher for E-cadherin and JAM-A, respectively) (Fig. 5A and 5B). These results indicate that, although cell morphology (cell growth in 2D layer) is very similar on ParC/PDMS and PS after 2 days of culture, ParC/PDMS should have a different impact on cell-cell communication (GAP and tight junctions) and to newly-synthesized ECM components (proteoglycans or collagens). Compared to PS, our data supports that ParC enabled enhanced cell-cell and cell-ECM interactions thanks to the high proliferation of HepG2 cells and the moderate increase in proteoglycans synthesis, as discussed before. Although formation of biliary pseudo-canaliculi must be analyzed by Scanning Electron Microscopy, we hope that ParC-coated substratum would provide a unique cellular microenvironment that may enhance cell-cell communication and functions of the biliary ways (cf. JAM-A).

Overall, these findings demonstrate that ParC/PDMS ameliorates cell-ECM (enhanced expression of α6- and β1-integrins) and cell-cell interactions (probable synthesis of biliary pseudo-canaliculi through an increase in expression of E-cadherin and JAM-A), compared to PS [54]. These findings strongly suggest that the similarity in roughness between ParC/PDMS and PS explains the comparable cell morphology, despite the existence of subtle differences regarding cell activities and the cellular interactions with the extracellular matrix. In accordance with the cell aggregation mechanisms previously described, we confirmed that the observed cell morphological modulations observed between ParC/PDMS and PDMS were induced by a significant difference in substrate roughness.

Such surface characteristics seem to initially induce passive self-organization and/or aggregation of collagen molecules, depending on the type of substratum. However, one cannot exclude the possibility of the involvement of surface chemistry in the attenuate changes in cellular behavior between HepG2 cells cultured on ParC/PDMS and PS substrata.

### Local Modulation of Cell Morphology: ParC/PDMS Micropatterning

We addressed the impacts of coating PDMS with ParC (morphology, cellular activities as well as cell-cell or cell-ECM interactions). We evaluated the feasibility of fabricating ParC/PDMS micropatterned substrata in order to locally control the cellular morphology in the case of HepG2 cells.

**Figure 6:**
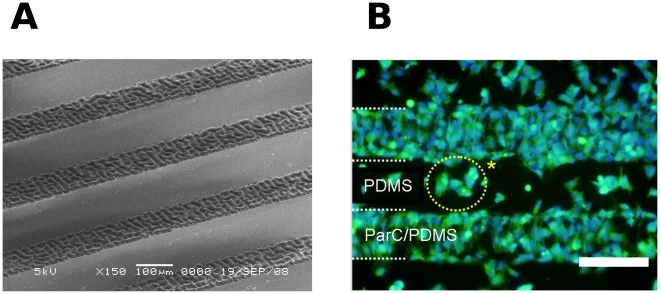
HepG2 cells on ParC/PDMS micropatterns. **A**) Scanning electron micrograph of ParC/PDMS micropatterns. Scale bar  = 100 µm. **B**) Fluorescent micrograph of HepG2 cells on ParC/PDMS micropatterns after two days of culture and stained with Calcein AM (green) and Hoechst (blue) fluorescent dyes. Scale bar  = 200 µm.

Local deposition of ParC was successfully accomplished using metal microstencils positioned in contact with PDMS. The obtained patterns of ParC/PDMS were then characterized by Scanning Electron Microscopy (SEM), which revealed clear local differences in surface roughness (Fig. 6A). After seeding HepG2 on these patterns, local differences in cell aggregates morphology (Calcein-Hoechst assay) were locally observed after two days of culture depending on the local nature of the substratum: small three-dimensional viable cell aggregates on PDMS and cell monolayer on ParC/PDMS (Fig. 6B).

These findings reveal the potential of ParC/PDMS micropatterns for locally modulating HepG2 cell culture growth and for controlling transition between two cellular phenotypes on the same platform. This local modulation is very encouraging for development of future tissue-engineered platforms that reproduce the vascular system of the hepatic lobule after advanced microsized patterning of ParC on PDMS.

### Conclusions

We first demonstrated the suitability of ParC substratum coating for long-term cell culture. Our findings show that the presence of ParC deposited on PDMS induces drastic differences in hepatocarcinoma cell aggregate morphogenesis, proliferation, and metabolic activity, as compared with PDMS alone. The observed disparities in cell activity and microenvironment were characterized in terms of cell phenotype, new ECM components synthesis, and the expression of transmembrane receptors implicated in cell-ECM, cell-cell adhesion and cell differentiation. ParC/PDMS substrata were also shown to induce higher cell differentiation compared to conventional PS substratum. We next showed that the observed changes in cell morphology and behavior between ParC/PDMS and PDMS alone were directly related to the substratum surface roughness, which may impact the supramolecular organization of adsorbed ECM. Finally, we achieved local modulation of HepG2 cell morphology and organization by randomly seeding cells on ParC/PDMS micropatterned substratum.

We strongly believe that specific control of ParC/PDMS micropattern dimensions by BioMEMS microfabrication will provide promising insight into how microscale ECM modifications directly impact cell morphology and activity. Further experiments will be dedicated to extend these studies to heterotypic cell cultures, such as hepatocyte-endothelial cell co-cultures, and to perform *in vitro* liver advanced tissue-engineered products that can mimic the *in vivo* arrangement of liver cells into a lobule with an “endothelial sinusoid vascularized-like” microfluidic network.

## Materials and Methods

### Substrata Preparation

Polydimethylsiloxane (PDMS) and parylene-C-coated PDMS (ParC/PDMS) were used as substratum materials and were compared to commercially available polystyrene (PS) substrata. First, a PDMS prepolymer (Silpot 184, Dow Corning) was mixed with a curing reagent at 1∶10 (v/v) and poured onto trifluoromethane (CHF_3_) plasma-treated silicon wafers. After degassing, the PDMS was cured at 70°C for 90 min. Flat PDMS membranes (thickness: 1.5 mm) were then obtained by peeling from the wafers. ParC/PDMS substrata were obtained after deposition of ParC onto PDMS membranes using a Low Pressure Chemical Vapor Deposition (LPCVD) system. Briefly, ParC was vaporized in a preliminary process zone at 175°C (P = 1 Torr) to form a gaseous dimer, di-para-xylene. The vapor resulting from this preliminary heating was then pyrolyzed in a second zone at 690°C (P = 0.5 Torr) to form para-xylene, a very reactive monomeric gas that could polymerize spontaneously and coat exposed PDMS substrata at room temperature (P = 0.1 Torr) at a predictable rate, resulting in a 1 µm- to 2 µm-thick coating under our experimental conditions. Since the deposition process was performed at room temperature, stresses induced by differential thermal expansion were avoided. Obtained PDMS and ParC/PDMS discs (6 mm in diameter) were inserted in standard PS Costar 24-well plates and were sequentially sterilized by oxygen (O_2_) plasma-treatment for 5 sec and with 70% ethanol for 10 min. Discs were then coated with 3 mg/mL collagen (Nitta Gelatin Co. Ltd.) overnight at room temperature before experimentation.

ParC/PDMS local micropatterns were obtained by depositing ParC onto PDMS through a 200 µm-thick nickel mask containing 200 µm-wide etched lines 300 µm apart. The metal stencil was adhered to the PDMS substrata using a sample holder and was mechanically removed after ParC deposition. The micro-structured ParC/PDMS substratum was then examined by Scanning Electron Microscopy (SEM, JEOL JSM-6600 LV microscope).

### Material Surface Characterization

Atomic force microscopy (AFM) measurements were performed at ambient temperature with a Nanoscope III Multimode AFM (Digital Instruments). All substrata were exposed to O_2_ plasma for 5 s and were then fixed in 3% glutaraldehyde overnight. After rinsing with water, samples were dried at 70°C for 12 h. Tapping-mode AFM was used to limit lateral deformation and to enhance image resolution. Independent measurements (n = 3) of 5×5 and 1×1 µm^2^ areas of each substratum were performed by randomly selecting a region. Root-mean-square roughness (R_rms_) of all substrata and the mean diameter of the collagen fibrils were evaluated using the image software provided by Digital Instruments. Contact angle measurements (n = 10) were performed by dispensing water drops (5–10 µL) on each substratum with a micropipette.

### Cell Culture

The HepG2 human hepatic carcinoma cell line (ATCC® Global Bio-resource Center™) was initially seeded at a density of approximately 4.5×10^4^ cells/cm^2^. Cells were then cultured for two days in DMEM (Gibco) containing 1 g/L glucose and supplemented with 25 mmol/L 4-(2-hydroxyethyl)-1-piperazineethanesulfonic acid, (HEPES, Dojindo), 100 U/mL penicillin, 100 µg/mL streptomycin, 1 µg/mL amphotericin B, 1% (v/v) non-essential amino acids (Gibco), and 10% (v/v) Fetal Bovine Serum (FBS, Gibco).

### Cell Viability and Morphology Analysis

Cells were incubated in 10 µmol/L Calcein-AM (Biosource) and 5 µmol/L Hoechst33342 (Sigma-Aldrich) solution for 30 min at 37°C, shielded from light in a humidified atmosphere of 5% CO_2_. Fluorescence emitted by Calcein-AM (λ_excitation_ = 494 nm, λ_emission_ = 517 nm) and Hoechst33342 (λ_excitation_ = 350 nm, λ_emission_ = 461 nm) was observed using an inverted fluorescent microscope (Olympus Corp.).

### Cell Topography by Scanning Electrochemical Microscopy

When a scanning microelectrode tip is moved laterally over a cell or field of cell culture, electrochemical data are recorded at multiple positions. A spatially-resolved image can then be constructed based on the local electrochemical properties of the area of interest.

We chose this technique to acquire topographic images of HepG2 cells cultured on different biomaterials. After two days of culture, HepG2 cells were fixed in 3% glutaraldehyde (Sigma-Aldrich) and then immersed in a ferricyanide (K_4_FeCN_6_) redox mediator solution (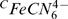 = 5 mmol/L in KCl 0.1 mol/L).

Cell culture topography was then recorded using FeCN_6_^4-^ oxidation potential (0.5 V vs. Ag/AgCl) since this hydrophilic redox mediator does not penetrate the cell membrane [33].

The scanning tip was first positioned above the cells of interest. When the electrode was placed far from the cells, a constant current of 4.6±0.2 nA was obtained, corresponding to the diffusion of the redox mediator in solution. When the electrode was moved toward the cell culture, the diffusion of the redox mediator to the electrode was partially blocked by the presence of the cells and a so-called negative feedback current was obtained, resulting in a decrease in the measured current. An approach curve was set to quantify the distance between the scanned cells and the scanning tip in accordance with theoretical equations. Under our experimental conditions, the tip approached the substratum until the measured current was about 3.5±0.2 nA, corresponding to a distance of 10–12 µm between the electrode and the cell monolayer. This initial distance between the tip and the first cell layer was found to be optimal for several reasons. First, the tip dimensions did not allow to position the tip directly at the substratum since the cell cultures were close to confluence. Second, to visualize cell aggregates above the cell monolayer without removing any of the adherent cells during scanning, the tip was placed at a distance that avoided any direct contact between the tip and the cells.

All measurements were performed using a scanning electrochemical microscope (SECM; HV-404, Hokuto Denko Co.) combined with a potentiostat (HA1010mM2B, Hokuto Denko Co.) comprising a 10 µm Pt-microdisk working electrode (d = 10 µm), a Pt counter electrode and a Ag/AgCl reference electrode (ALS Co.). All images were acquired in the constant height mode within 1 h (scan rate: 10 µm/s; resolution: 40×40 pixels; scan area: 400×400 µm).

### Cell growth, proliferation and functionality

The HepG2 cells were detached from the substrata by conventional trypination after 4 hours (day 0), 2 days, 8 days and 14 days of cell culture. Cell proliferation was then quantified after staining with trypan blue and counting viable cells with a hemocytometer. Cell density was expressed as the number of HepG2 cells per centimeter square. Several samples were counted for each substratum to perform a valid statistical study on cell density and to include probable heterogeneity between the samples.

Glucose consumption was measured using a glucose analyzer (Glucose Analyzer 2, Beckman Instruments Inc.). Human albumin levels were quantified in culture supernatants using a sandwich enzyme-linked immunosorbent assay (ELISA) with goat anti-human albumin- and horseradish peroxidase (HRP)-conjugated antibodies (Bethyl Laboratories Inc., TX). Absorbance was measured at 450 nm using a multi-well microplate reader (MPR A4i, Tosoh). Albumin and glucose concentration values were then normalized to 10^6^ cells so that the absolute concentrations could be evaluated.

Proteoglycans' levels and total collagen were analyzed separately. Cells were washed with phosphate buffered saline (PBS) and fixed in 3% glutaraldehyde solution before staining. For collagen observation, immortalized cells were sequentially exposed, at room temperature, to 0.1% w/v Sirius red solution (Sigma-Aldrich) for 1 h and 0.01 mol/L hydrochloric acid (HCl) for 2 min. The presence of proteoglycans was assessed by exposing the cells at room temperature to 0.1% w/v Alcian blue in acetate (Sigma-Aldrich) for 2 h and 0.1% w/v Nuclear Fast Red in 5% aluminum sulfate (Sigma-Aldrich) for 10 min. After washing the stained samples, cell-synthesized ECM components were analyzed under a photonic microscope (Olympus Corp.).

Cell activity measurements were performed by taking into account the cell density for each substratum, and were then given per million of cell.

### Cell-Cell and Cell-ECM Interaction Analysis

Integrins, E-cadherin and Junctional Adhesion Molecule (JAM)-A levels were evaluated separately by fluorescence. After washing with PBS, cells were fixed and permeabilized in 3% v/v glutaraldehyde solution and 0.3% v/v Triton-X (Wako), respectively. Cells were then sequentially incubated with a blocking buffer (5% w/v bovine serum albumin (BSA) in PBS) for 2 h at room temperature to avoid any non-specific protein/antibody reactivity, and then in shielded from light in a humidified atmosphere of 5% CO_2_ in the presence of fluorescein (FITC)-conjugated antibodies (mouse monoclonal anti-human β1-Integrin (Biosource), mouse monoclonal anti-human α6-Integrin (Biosource), monoclonal anti-E-cadherin (Sigma)) diluted at 1∶100 or 1∶125 in PBS containing 1% BSA for 2 h at 37°C. In the case of JAM-A, cells were sequentially incubated for 2 h at 37°C in the presence of a rabbit polyclonal primary anti-JAM-A antibody (Zymed Laboratories Inc) diluted at 1∶250 and then shielded from light in a humidified atmosphere of 5% CO_2_ in the presence of fluorescein (FITC)-conjugated secondary anti-IgG antibody (Sigma) diluted at 1∶10000. After three PBS washes, each substratum was observed using an inverted fluorescent microscope (Olympus Corp.). Fluorescence intensity per surface unit was quantified using NIH Image software (Image J, http://www.rsb.info.nih.gov). As for cell activities, the obtained values were normalized following the obtained cell density for each substratum and then expressed per 10^6^cells.

### Statistical Analysis

Data were expressed as mean ± standard deviation (SD) of three to nine values obtained from three separate experiments. Comparisons were made by ANOVA, followed by Tuckey's protected least significant difference test using R_Commander statistical software (Rcmdr package, http://www.r-project.org/). Statistically significant difference from PDMS was indicated as *, p<0.05 and from PS as #, p<0.05.

## Supporting Information

Figure S1 SECM measurement principle. A) Scheme of SECM experimental setup and B) experimental approach curve obtained for the used scanning tip (Pt, d = 10 µm) and principle of cell topography by SECM (I_T_  =  recorded tip current, I_∞_  =  current intensity obtained in solution).
(0.24 MB EPS)
